# Carbon Ion Radiotherapy for Locally Recurrent Rectal Cancer of Patients with Prior Pelvic Irradiation

**DOI:** 10.1245/s10434-021-10876-4

**Published:** 2021-10-18

**Authors:** Shigeru Yamada, Hirotoshi Takiyama, Yuka Isozaki, Makoto Shinoto, Daniel K. Ebner, Masashi Koto, Hiroshi Tsuji, Hideaki Miyauchi, Mitsugu Sekimoto, Hideki Ueno, Michio Itabashi, Masataka Ikeda, Hisahiro Matsubara, for the Working Group on Locally Recurrent Rectal Cancer

**Affiliations:** 1 QST Hospital, National Institutes for Quantum and Radiological Science and Technology, Chiba, Japan; 2 Graduate School of Medicine, Chiba University, Chiba, Japan; 3 Kansai Medical University Hospital, Osaka, Japan; 4 National Defense Medical College, Saitama, Japan; 5 Tokyo Womens Medical University, Tokyo, Japan; 6 Hyogo College of Medicine, Hyogo, Japan

## Abstract

**Background:**

This study aimed to assess the safety and efficacy of carbon-ion radiotherapy (CIRT) for salvage of previously X-ray-irradiated (XRT) locally recurrent rectal cancer (LRRC).

**Methods:**

Between September 2005 and December 2017, 77 patients with LRRC were treated with CIRT re-irradiation. All the patients had received prior XRT with a median dose of 50.0 Gy (range 20–74 Gy), principally for neoadjuvant or adjuvant recurrence prophylaxis in 34 patients and for recurrence in 43 patients. The total CIRT dose of 70.4 Gy (RBE) (gray relative biologic effectiveness) was administered in 16 fixed fractions during 4 weeks (4.4 Gy [RBE] per fraction).

**Results:**

All the patients completed the scheduled treatment course. None of the patients received resection after CIRT. Acute grade 3 toxicities occurred for eight patients (10 %), including five grade 3 pelvic infections (2 involving pain and 1 involving neuropathy). Late grade 3 toxicities occurred for 16 patients (21 %): 13 with late grade 3 pelvic infections, 9 with gastrointestinal toxicity, 1 with skin toxicity, 2 with pain, and 4 with neuropathy. No grade 4+ toxicity was noted. The overall local control rates (infield + out-of-field recurrence) were 69 % at 3 years and 62 % at 5 years. In the planning target volume (PTV), the infield recurrence rates were 90 % and 87 % respectively. The control rates for regional recurrence were 85 % at 3 years and 81 % at 5 years. The median overall survival time was 47 months. The survival rates were 61 % at 3 years and 38 % at 5 years.

**Conclusion:**

Carbon-ion re-irradiation of previously X-ray-irradiated locally recurrent rectal cancer appears to be safe and effective, providing good local control and survival advantage without unacceptable morbidity.

In Japan, 51,238 patients had rectal cancer in 2017, with 15,244 deaths in 2018.^1^ Locally recurrent rectal cancer (LRRC) occurs for 10 % to 20 % of patients receiving curative resection of rectal cancer.^2–4^ Although the use of adjunctive pre- or postoperative radiation/chemoradiation therapy has reduced the incidence of LRRC, 4 % to 13 % of patients still experience recurrence in the pelvis.^5–7^ Quality of life can be severely affected by LRRC, leading to severe pain, concomitant neurologic disorders, pelvic infection, bleeding, and bowel obstruction.

The only curative treatment for LRRC after X-ray radiotherapy (XRT) is resection. However, resection after XRT is highly invasive, and the incidence of postoperative normal tissue complications is high.^8^ Repeated surgery for recurrent tumors is complicated, not only by a loss of normal anatomic tissue structure due to adhesions but also by additionally significant fibrosis after irradiation.^9^

Chemotherapy has been developed in recent years, but the response rate of local recurrence can be as low as 10 % compared with that of distant metastasis.^10^ Therefore, salvage often is performed with radiation therapy. However, because surrounding critical organs such as the small intestine, colon, and bladder may have already received doses near organ tolerance doses during the primary treatment, re-irradiation is associated with a comparatively higher risk of acute and late toxicity.

In terms of radiotherapeutic optimization, the lethality of the dose delivered to the target tumor must be balanced with the toxicity of irradiating surrounding normal tissue. The carbon-ion beam possesses unique physical and biologic properties that enhance its usage in this regard,^11,12^ offering improved dose localization, allowing greater concentration of dose within target tissues as well as enhanced biological efficacy due to its nature as a high-linear-energy-transfer (LET) radiation. These properties include the induction of more cell cycle- and oxygenation-independent, irreversible cell damage than is observed with low-LET radiation such as XRT.

Favorable results with CIRT in the treatment of LRRC have previously been demonstrated. For patients with LRRC who received CIRT, the 5-year rates were 88 % for local control (LC) and 59 % for overall survival (OS).^13^ Shinoto et al.^14^ conducted a multi-center retrospective evaluation of 224 LRRC patients treated with CIRT, reporting a 5-year OS rate of 73 % and an LC rate of 88 %. It was hypothesized that CIRT may offer efficacy similar to that of a re-irradiation method for LRRC. To improve long-term local control and OS of LRRC, LRRC patients with prior pelvic XRT and recurrent disease have been treated at our institution since 2006.

This study aimed to determine the rates for acute and late toxicity, local control, and OS for 77 patients who had LRRC treated with carbon-ion re-irradiation at a single institution. Furthermore, potential factors associated with toxicity, local control, and survival were evaluated.

## Patients and Methods

Institutional review board approval was received for retrospective evaluation of 77 LRRC patients treated with CIRT re-irradiation between September 2005 and February 2017. The study was conducted with the approval of the Institutional Review Board (19-008) and performed in accordance with the Declaration of Helsinki. All the patients provided informed consent for use the data from their medical records.

### Patient Eligibility

Patients were eligible for this study if they had local recurrence of rectal cancer without distant metastasis verified by computed tomography (CT), magnetic resonance imaging (MRI), and C11-methionine positron emission tomography (PET) imaging; had rectal adenocarcinoma; had a distance than 3 mm from the recurrent tumor edge to the bowel, bladder, and urethra in both prone and supine positioning; had a radiographically measurable tumor (*t* ≤ 15 cm); and had a Karnofsky performance score of 70 or higher. Patients were excluded if they had received chemotherapy within 4 weeks of CIRT, had received prior radiotherapy to the same target site, or had another primary malignancy. Before patient registration, a full history with a physical exam was performed, including MRI, CT, and PET, allowing characterization of disease status, extent, and size.

### Carbon Ion Radiotherapy

The Heavy Ion Medical Accelerator in Chiba (HIMAC) is the world’s first heavy ion accelerator complex dedicated to medical use in a hospital environment. The features of the accelerator and carbon ion beam have been described previously.^13^

The patients were positioned in customized cradles (Moldcare; Alcare, Tokyo, Japan) and immobilized with a low-temperature thermoplastic shell (Shellfitter; Kuraray Co, Ltd, Osaka, Japan). A set of 2-mm-thick CT images was taken for treatment planning with the immobilization devices. Respiratory gating of both the CT acquisition and the therapy was performed.

Three-dimensional (3D) treatment planning was performed using the in-house HIPLAN (National Institute of Radiological Sciences, Chiba, Japan) and Xio-N (ELEKTA, Stockholm, Sweden; Mitsubishi Electric, Tokyo, Japan) planning software. The position was collated by matching the bone structure visualized by the treatment plan CT with the bone structure acquired in the treatment room immediately before irradiation as much as possible. The CIRT treatment was given once daily, 4 days a week (Tuesday to Friday) for a fixed 16 fractions in 4 weeks. The dose was set at 70.4 Gy (RBE) (4.4 Gy [RBE] per fraction), as derived from the previous phase 1/2 trial of CIRT for pelvic recurrence of rectal cancer.

Two to five irregularly shaped ports (median, 3 ports) were used for the CIRT treatment. The target volume definition was determined on the basis of contrast-enhanced CT, MRI, and PET imaging. The gross tumor volume (GTV) was contoured as the macroscopic tumor visible on imaging, and the clinical target volume (CTV) was determined by adding a 5-mm margin to the GTV. The planning target volume (PTV) had a margin of 3 to 5 mm added around the CTV.

**Fig. 1.**
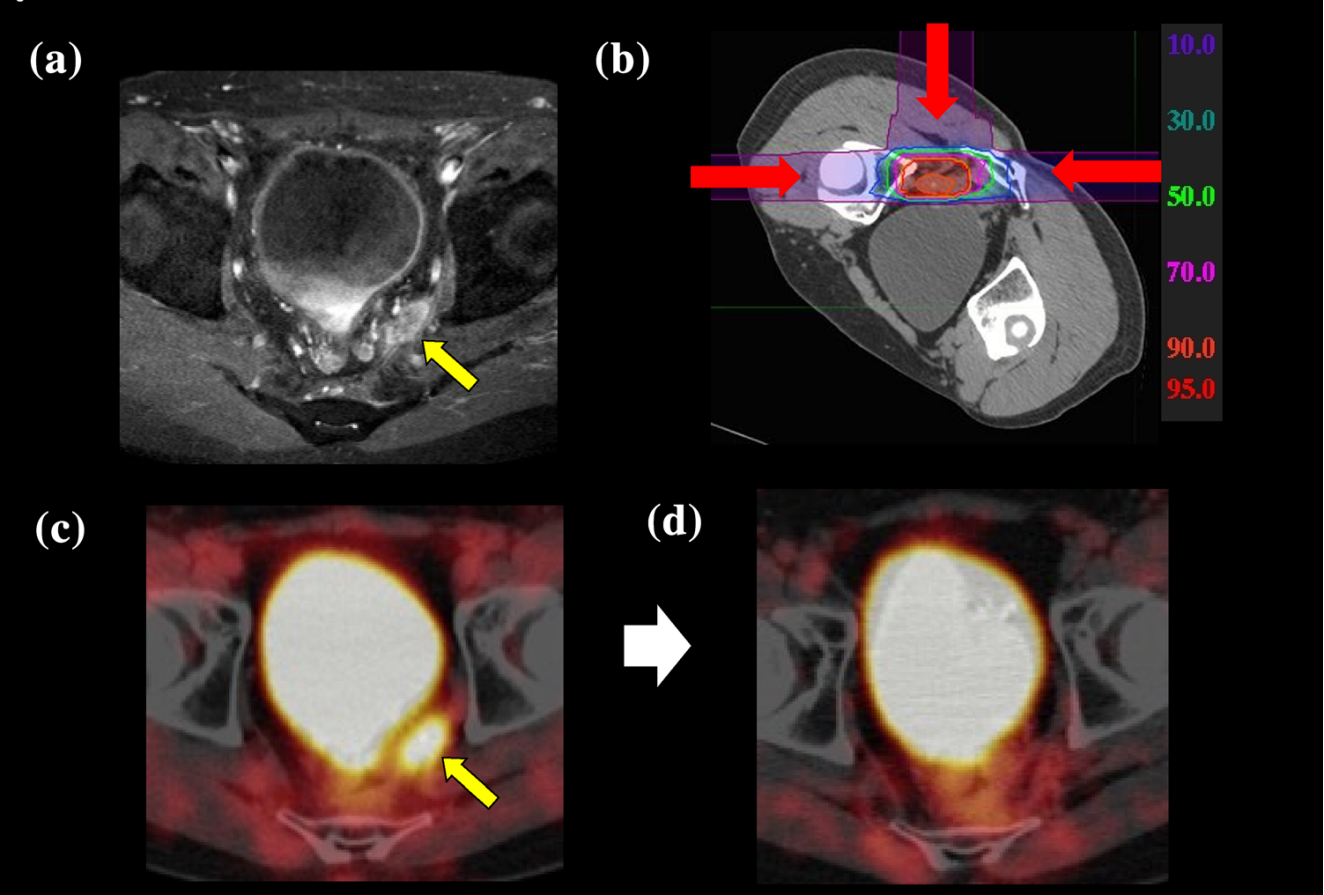
This patient had received 40 Gy X-ray treatment 17 months before carbon-ion radiotherapy (CIRT). **a** T1 magnetic resonance imaging before treatment showing a tumor mass in the presacral space (*yellow arrow*). **b** Depth-dose distribution of the carbon ion beam in recurrent rectal cancer (*red line:* 90 % isodose of the prescribed dose). **c** Positron emission tomography (PET) imaging before treatment. **d** The PET 48 months after treatment demonstrating disappearance of the left-side wall mass. At this writing, the patient is alive 8 years after CIRT with no signs of recurrence.

The dose constraints for the D2cc of the intestine and bladder were set at 50 Gy (RBE) and 60 Gy (RBE) in 16 fractions, respectively, and at 60 Gy (RBE) and 70 Gy (RBE) when combined with the dose distribution of the previous XRT, based on prior evaluations by Okonogi et al.^15^ and Kim et al.^16^ Examples of PTV with isodose distributions are shown in Fig. 1.

### Toxicity Criteria

Acute and late toxicities were defined according to the National Cancer Institute–Common Toxicity Criteria version 5.0 and determined through retrospective chart review. Acute toxicity was defined as toxicity persisting within 90 days after the completion of CIRT treatment. Scores for late toxicities were those for the highest late toxicities observed 3 months or later after CIRT.

### Tumor Response and Local Control Criteria

After treatment, CT or MRI was performed every 3 months for 2 years, then at 6-month intervals thereafter. Local recurrence was defined as evidence of tumor volume enlargement or the appearance of a new lesion in or around the PTV.

Recurrent tumor was defined as an infield recurrence when its center was inside the PTV and as an out-of-field recurrence when its center was outside the PTV. Regional recurrence was defined as a new tumor in the pelvic region other than the PTV. Progression-free survival (PFS) was defined as the time from the start of CIRT to the earliest signs of disease progression or death from any cause. Overall survival was defined as the time from CIRT to death from any cause and was censored at the date of the last follow-up visit for surviving patients.

### Follow-Up Evaluation

All the patients were seen on a regular basis during the follow-up period. The initial evaluation of tumors using CT, MRI, and PET scans was performed within 1 month after the completion of CIRT. Thereafter, the patients were followed up by CT or MRI every 1 or 2 months for the next 6 months. Then the intervals between imaging and follow-up visits were extended by 3 to 6 months. After the initial evaluation, PET was not performed regularly.

### Statistical Analysis

For statistical analysis, JMP (SAS Institute Inc.) was used. Survival and control curves were generated by the Kaplan–Meier method.

## Results

### Patient Characteristics

From September 2005 to February 2017, 77 patients with LRRC and a history of pelvic radiotherapy with X-ray underwent re-irradiation with CIRT at a single institution. All the patients were confirmed as having LRRC without distant metastasis by CT, MRI, and fluorodeoxyglucose (FDG)-PET examination, and as having adenocarcinoma of the rectum. The hospital and radiotherapy records of these patients were reviewed. All the patients signed an informed consent form approved by the NIRS (now QST) review board.

The characteristics of the patients are summarized in Table 1. The median age was 60 years (range, 37–76 years). The origin of 29 relapses was in the presacral region, with 23 relapses in the pelvic sidewalls and 15 relapses in the perineal region.

**Table 1:** Patient characteristics

	Total
Number of patients	77
Median, years (range)	60 (37–76)
Female/male	54/23
*Surgical approach*	
Abdominoperineal excision	26
Low anterior resection	43
Hartmann’s resection	7
Total Pelvic exenteration	1
*Histologic diagnosis*	
Adenocarcinoma, well differentiated	26
Medium differentiated	32
Poorly differentiated	6
Unknown differentiation	9
Mucinous	2
Unclear/combination	2
Post surgical recurrence median month (Range)	49.9 (13.0–157.0)
*Purpose of previous RT*	
Neoadjuvant	27
Adjuvant	9
Recurrence treatment	43
Previous RT median dose (range)	50 Gy (20–74)
*Site of recurrence*	
Presacral	29
Side wall	23
Perineal	15
Recurrent tumor median size (range)	40 mm (14–110)
Surgical spacer placement	13

*Two patients received both neoadjuvant and adjuvant ratiotherapy.

All the patients had received prior XRT. The median dose of the previous radiotherapy was 50 Gy (range 20–74 Gy), with prior radiation given as neoadjuvant or adjuvant recurrence prophylaxis for 34 patients and as treatment of recurrence in 43 patients. The median interval from the time of surgery to the time of re-irradiation therapy was 50 months (range 13–157 months).

At this writing, none of the patients have undergone resection after CIRT.

Before CIRT, four patients had received spacer implantation by open surgery to create an appropriate distance between the intestine and the tumor, with the spacer fixed tightly to the peritoneum. In 11 cases, polytetrafluoroethylene (PTFE) prostheses were placed, and in 1 case, an omental flap was used to create space between the tumor and the intestine.^17^

### Toxicity

The toxicities in the 77 lesions receiving re-irradiation with CIRT are listed in Table 2. Skin changes by treatment were relatively few and mild in these patients. Eight acute grade 3 toxicities were observed (Table 2). Five grade-3 pelvic infections occurred (2 involving pain and 1 involving neuropathy). Of the five grade 3 pelvic infections, two were skin and fistula formations before CIRT, two were spacer insertion infections, and the last was an intratumoral infection. In 16 patients (21 %), 29 late grade 3 toxicities occurred. There were 13 pelvic infections, 9 gastrointestinal toxicities, 1 skin reaction, 2 cases of pain, and 4 cases of neuropathy. Of the 13 grade 3 pelvic infections, 5 were due to tumoral perforation of gastrointestinal tracts, 4 were due to tumoral perforation of the skin, and 1 was due to tumoral perforation of the vagina.

**Table 2:** Acute and late toxicities

	Acute						Late					
	G0	G1	G2	G3	G4	Total	G0	G1	G2	G3	G4	Total
Skin	23	51	3	0	0	77	25	48	3	1	0	77
GI	52	9	6	0	0	77	66	1	1	9	0	77
GU	70	2	5	0	0	77	73	0	4	0	0	77
Infection	64	5	3	5	0	77	60	1	3	13	0	77
Pain	36	23	16	2	0	77	35	24	16	2	0	77
Neuropathy	39	28	9	1	0	77	33	23	17	4	0	77

*GI* gastrointestinal tract; *GU* genitourinary tract

### Tumor Response

**Fig. 2.**
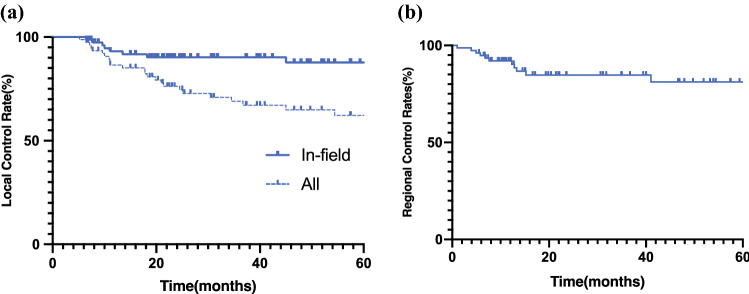
Kaplan–Meier estimates of local and regional control rates for patients treated with carbon-ion re-irradiation. **a** The local control rates (all: infield + out-of-field recurrence) were 69 % (95 % CI 56–79 %) at 3 years and 62 % (95 % CI 51–73 %) at 5 years. In the PTV, the infield recurrence rates were 90 % (95 % CI 81–95 %) at 3 years and 87 % (95 % CI 76–93 %) at 5 years. **b** The control rates for regional recurrence were 85 % (95 % CI 73–92 %) at 3 years and 81 % (67 % CI 81–90 %) at 5 years. *CI* confidence interval; *PTV* planning target volume

The median follow-up duration was 45 months (range 7–159 months) for all the patients, and 72 months (range 28–159 months) for the surviving patients. The local recurrence analysis is presented in Fig. 2. Local recurrence was observed in 26 of the 77 lesions (8 cases of infield recurrence and 18 cases of out-of-field recurrence). The overall local control rate (infield + out-of-field recurrence) was 69 % (95 % confidence interval [CI] 56–79 %) at 3 years, and 62 % (95 % CI 51–73 %) at 5 years. The rate of infield recurrence within the PTV specifically was 90 % (95 % CI 81–95 %) at 3 years and 87 % (95 % CI 76–93 %) at 5 years. The control rate for regional recurrence was 85 % (95 % CI 73–92 %) at 3 years and 81 % (67 % CI 81–90 %) at 5 years.

### Survival

**Fig. 3.**
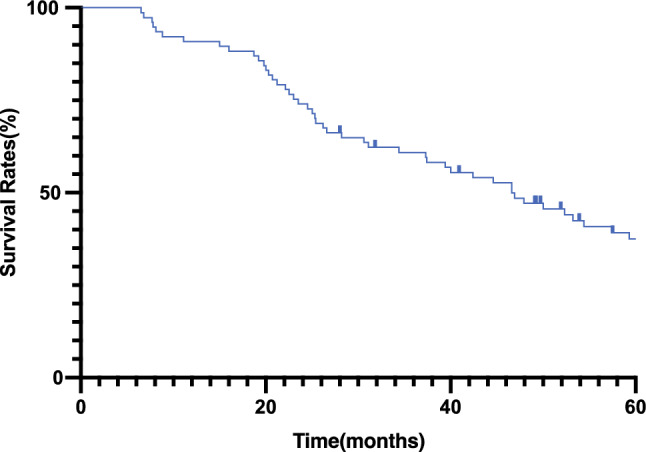
Kaplan–Meier estimates of overall survival for patients treated with carbon-ion re-irradiation.

**Fig. 4.**
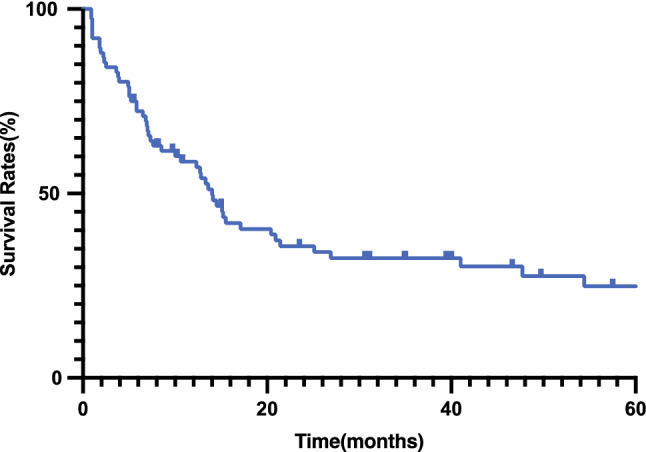
Kaplan–Meier estimates of progression-free survival for patients treated with carbon-ion re-irradiation.

The OS and disease-free survival (DFS) estimates for the 77 analyzed patients receiving re-irradiation with CIRT are shown in Fig. 3. The median OS time was 47 months. The OS rate was 61 % (95 % CI 49–71 %) at 3 years and 38 % (95 % CI 26 –49 %) at 5 years. The PFS rate was 33 % (95 % CI 22–44 %) at 3 years and 25 % (95 % CI 15–37 %) at 5 years (Fig. 4).

## Discussion

In the 77 patients, CIRT re-irradiation for LRRC was well tolerated, with positive patient outcomes. These results are encouraging because the patients in this population were not eligible for surgical resection and had few options for definitive therapy. The OS rate appears promising, with a 3-year survival rate of 61 %, a 5-year survival rate of 38 %, and a median OS time of 47 months, even for patients who did not undergo surgical resection.

The literature reports a 3-year survival rate of 20 % to 27 % for LRRC in patients who had prior pelvic irradiation treated with conventional radiotherapy and were unable to undergo post-irradiation surgery, as well as a survival rate of 60 % to 67 % for those able to receive surgery.^18–20^ Lee et al.^21^ reported a meta-analysis of re-irradiation for postoperative recurrence of rectal cancer, with rates of 85.9 % at 1 year, 71.8 % at 2 years, and 51.7 % at 3 years for the surgery group, and corresponding rates of 63.5 %, 34.2 %, and 23.8 % for the nonsurgical candidates, indicating significantly higher survival rates for those receiving surgery. Indeed, radical resection appears to be the most significant measure of improved survival for patients with LRRC,^22^ suggesting that a high local control rate correlates with survival prolongation.

Unfortunately, curative surgery is possible only for a limited number of cases, necessitating radiation therapy for improvement in local control. However, due to the difficulty of overlapping conventional radiotherapy, with prior irradiation fields and general concern for toxicity, the re-irradiation dose often is limited to 30 to 40 Gy.^23^ At this dose, an anti-tumor effect compatible with a complete response or enhanced survival cannot be expected. Koom et al.^24^ showed that patients who received high-dose re-irradiation (>50 Gy) with a conventional 2-Gy fractionation scheme had a significantly higher infield PFS than patients who received a low-dose re-irradiation (≤50 Gy).

Hypofractionated radiation techniques such as intensity-modulated radiotherapy (IMRT) and stereotactic body radiotherapy (SBRT) can reduce gastrointestinal (GI) toxicity compared with conventionally fractionated radiotherapy (RT) through sparing of normal tissue,^25^ and may improve overall outcome. Although IMRT may offer enhanced target dose delivery, it suffers from a wider low-dose area, and translation of the technique into a clinical outcome for re-irradiation remains under study.

Fady et al.^26^ evaluated IMRT re-irradiation (median dose, 30.4 Gy [range 27–40 Gy] in 15 to 22 fractions) for 31 cases of rectal cancer recurrence after surgery. The median OS was 21.9 months, and the 1-year survival rate was 66.7 % for the patients who had surgical resection versus 58.7 % for those who did not.

Susko et al.,^27^ analyzed differences in toxicity and tumor control among RT methods and found that the re-irradiation technique (2D/3D, IMRT, intraoperative radiation therapy [IORT]) was not associated with OS differences (*p* = 0.46). Meanwhile, SBRT can deliver higher doses to tumors due to a reduced mechanical error margin, with less normal tissue damage.

Dagoglu et al.^28^ evaluated SBRT re-irradiation for 22 cases of rectal cancer recurrence after surgery that delivered a mean dose of 25 Gy in five fractions. The median survival was 40 months. The 3-year survival rate was 59.3 %, and the local control rate was 85.9 %.

Kim et al.^29^ reported on 23 patients treated with 30 to 51 Gy delivered in three fractions, showing a 4-year OS rate of 24.9 % and a local control rate of 74.3 %. For re-irradiation, SBRT is expected to be a highly effective treatment method, but the number of reports is small, and the number of cases reported to date is limited.^30^ More study is needed, particularly with regard to combination methods incorporating systemic agents.

Carbon ions have potential advantages over photons in providing a better physical dose distribution, with lateral scattering less than with proton-beam radiotherapies.^31^ For recurrent patients who had not previously received radiation therapy, CIRT had a 5-year local control rate of 88 % (95 % CI 80–93 %), whereas CIRT re-irradiation demonstrated a control rate of 62 % (95 % CI 51–73 %), although notably with a 5-year infield local control rate within the PTV of 93 % (95 % CI 86–96 %) for patients without previous irradiation and 87 % (95 % CI 76–93 %) for those with previous irradiation, showing no significant difference. Given that the limitation in control was noted to be outside of the PTV, one method for improving local control may involve broadening the PTV to better capture these locoregional disease sites. Inherent differences in systemic dissemination of disease between patients receiving adjuvant treatment and those undergoing CIRT re-irradiation also may be considered.

Acute grade 3 toxicities were observed in eight patients (10 %) and late grade 3 toxicities in 16 patients (21 %). According to a systematic review reported by Kim et al.,^21^ the grade 3 acute and late complication rates in conventional radiotherapy were respectively 11.7 % and 25.5 %. The number of grade 3 or higher CIRT toxicities were therefore similar to those in other reports. With regard to the five acute grade 3 pelvic infections, two were skin and fistula formations occurring before CIRT, and two were spacer insertion infections. Of the 12 patients with late grade 3 infections, five cases were a result of spacer placement, and four cases involved skin and fistula formations before CIRT. Collectively, these 5 and 12 acute and late toxicities likely were unrelated to CIRT. Excluding the spacer, the relatively high rate of gastrointestinal toxicities noted may have been due to a high dose delivered to GI tracts surrounding the tumor using the previous-generation passive-beam irradiation method.

Currently, 3D respiration-gated scanning irradiation with a rotating gantry are used, likely allowing further reduction in the normal tissue dose, which may further reduce the rate of adverse events. Evaluation of this is in progress. Similarly, use of a bioabsorbant polyglycolic acid spacer was approved in July 2020, allowing a sufficient margin for CIRT, even in cases with a short distance between the tumor and the gastrointestinal tract or bladder. Ongoing optimization of spacer technology may allow for further reduced toxicity.^32^

Our data suggest that CIRT is feasible and tolerable for patients with favorably located tumors (>3 mm from the nearest luminal organ) and where modern CIRT equipment allows for conformal avoidance of dose-limiting organs. Under these conditions, the number of target cases that can be analyzed is limited. Nonetheless, the 3-year OS rate for CIRT is threefold higher than for XRT, and comparable with the results of surgical resection. Collectively, this may represent an important advance in improving the outcome of treatment for LAPC patients.

In summary, CIRT treatment of LRRC in patients with previous pelvic irradiation demonstrated effective local control with acceptable levels of acute severe toxicity and may serve as promising alternative to surgery for patients whose treatment is technically difficult. Late toxicity remains comparable with that of other radiation therapies, and incorporation of the CIRT scanning irradiation method, rotating gantry, and in-house multi-ion irradiation method^33^ is expected to yield a further improved therapeutic ratio. Further evaluation continues.
